# Integrating expert opinion with clinical trial data to extrapolate long-term survival: a case study of CAR-T therapy for children and young adults with relapsed or refractory acute lymphoblastic leukemia

**DOI:** 10.1186/s12874-019-0823-8

**Published:** 2019-09-02

**Authors:** Shannon Cope, Dieter Ayers, Jie Zhang, Katharine Batt, Jeroen P. Jansen

**Affiliations:** 1 Precision Xtract, 1505 West 2nd Avenue, Suite 300, Vancouver, BC V6H 3Y4 Canada; 2 Global Oncology Strategy and Business Dev, Novartis Pharmaceuticals Corporation, 1 Health Plaza, East Hanover, NJ 07936 USA; 3 Wake Forest Baptist Medical Center, 1 Medical Center Blvd, Winston-Salem, NC 27157 USA; 4 Precision Xtract, 555 12th Street, Suite 250, Oakland, CA 94607 USA

**Keywords:** Survival, Expert elicitation, Pediatric, Leukemia, Tisagenlecleucel

## Abstract

**Background:**

Long-term clinical outcomes are necessary to assess the cost-effectiveness of new treatments over a lifetime horizon. Without long-term clinical trial data, current practice to extrapolate survival beyond the trial period involves fitting alternative parametric models to the observed survival. Choosing the most appropriate model is based on how well each model fits to the observed data. Supplementing trial data with feedback from experts may improve the plausibility of survival extrapolations. We demonstrate the feasibility of formally integrating long-term survival estimates from experts with empirical clinical trial data to provide more credible extrapolated survival curves.

**Methods:**

The case study involved relapsed or refractory B-cell pediatric and young adult acute lymphoblastic leukemia (r/r pALL) regarding long-term survival for tisagenlecleucel (chimeric antigen receptor T-cell [CAR-T]) with evidence from the phase II ELIANA trial. Seven pediatric oncologists and hematologists experienced with CAR-T therapies were recruited. Relevant evidence regarding r/r pALL and tisagenlecleucel provided a common basis for expert judgments. Survival rates and related uncertainty at 2, 3, 4, and 5 years were elicited from experts using a web-based application adapted from Sheffield Elicitation Framework. Estimates from each expert were combined with observed data using time-to-event parametric models that accounted for experts’ uncertainty, producing an overall distribution of survival over time. These results were validated based on longer term follow-up (median duration 24.2 months) from ELIANA following the elicitation.

**Results:**

Extrapolated survival curves based on ELIANA trial without expert information were highly uncertain, differing substantially depending on the model choice. Survival estimates between 2 to 5 years from individual experts varied with a fair amount of uncertainty. However, incorporating expert estimates improved the precision in the extrapolated survival curves. Predictions from a Gompertz model, which experts believed was most appropriate, suggested that more than half of the ELIANA patients treated with tisagenlecleucel will survive up to 5 years. Expert estimates at 24 months were validated by longer follow-up.

**Conclusions:**

This study provides an example of how expert opinion can be elicited and synthesized with observed survival data using a transparent and formal procedure, capturing expert uncertainty, and ensuring projected long-term survival is clinically plausible.

**Electronic supplementary material:**

The online version of this article (10.1186/s12874-019-0823-8) contains supplementary material, which is available to authorized users.

## Introduction

Decision-makers need to understand long-term clinical outcomes to assess cost-effectiveness of new treatments over a lifetime horizon. In the absence of long-term data from clinical trials, current practice to extrapolate observed survival data beyond the clinical trial follow-up period typically involves fitting alternative parametric models to the observed survival. The choice regarding which parametric model is most appropriate is often driven by how well each model fits to the observed data [1]. However, models with a similar fit to the observed data may yield dramatically different estimates of long-term survival due to differences in the extrapolation; this is especially the case with limited follow-up. Despite the sensitivity of cost-effectiveness estimates to extrapolation, conventional cost-effectiveness models typically do not explicitly ‘consider external long-term validity’ [2] of extrapolations from clinical data. The National Institute for Health and Care Excellence (NICE) recommends that any extrapolation should consider ‘both clinical and biological plausibility of the inferred outcome as well as its coherence with external data sources’; however they do not specify any methodologies to accomplish this.

One way to potentially improve the plausibility of survival extrapolations is to supplement the clinical trial data with feedback from clinical experts. However, evidence from experts’ opinion is rarely incorporated into cost-effectiveness analyses in a formal manner [3–5]. At best, one to two clinicians are consulted to provide an opinion about the most ‘realistic’ model to extrapolate the observed data. Experts are almost never asked about their estimations of long-term survival prior to being presented with alternative model extrapolations. Moreover, they are not formally asked to express the uncertainty in their estimates. Recently, Jackson et al. (2017) outlined the potential benefits of formally eliciting long-term survival estimates based on expert opinion as an alternative to post-hoc questions as to which statistical model is most appropriate, but indicated that additional research is needed [2].

The aim of this paper is to demonstrate the feasibility of systematically integrating long-term survival estimates obtained from a formal expert elicitation study with empirical clinical trial data in an attempt to provide more credible extrapolated survival curves through a case study.

## Motivating case study

Pediatric acute lymphoblastic leukemia (pALL) is the most common pediatric cancer in the United States and represents about one quarter of cancer diagnoses among children under the age of 15 years [6]. Among pediatric and young adult patients with B-cell ALL, less than 10–30% of patients who have had multiple relapses or become treatment refractory remain disease-free at five years [7–9]. Tisagenlecleucel (Kymriah®) is the first chimeric antigen receptor T-cell (CAR-T) therapy approved by Food and Drug Administration (FDA) for the treatment of pediatric and young adult patients (up to 25 years) with B-cell precursor ALL that is refractory or in second or later relapse.^1^ Tisagenlecleucel has a novel mechanism of action, involving autologous T cells genetically modified with a CAR to target CD19 on the surface of malignant B cells [10]. In their curative intent model, Hettle et al. [11] evaluated the cost-effectiveness of tisagenlecleucel, based on 15 months of survival data from a phase I/II single-center trial CHP959 (NCT01626495) based in the United States [10]. Their analysis highlighted the dramatic differences in the expected survival (i.e. area under the curve) depending on the statistical model used for extrapolation of the empirical survival data. Following the evaluation by Hettle et al., a global multicenter ELIANA trial (NCT02435849) for tisagenlecleucel was published [12], reporting survival data out to 1.5 years. This pivotal trial provides a larger and more representative patient sample than the phase I/II study CHP959. However, the uncertainty regarding long-term survival remains.

In the context of relapsed or refractory (r/r) pALL, the challenge of extrapolating survival data is compounded by the young age of patients, differences among patients in terms of relapse or refractory disease and treatment history, limited evidence regarding best supportive care, the absence of randomized controlled trials for tisagenlecleucel (i.e. single-arm trials only), the possibility for curing patients, and the innovative nature of the new treatment. Given the high degree of uncertainty regarding long-term survival in this population, a method to integrate estimates from experienced clinicians may provide more value than the traditional curve fitting process based solely on survival observed from the clinical trial.

## Methodology

Based on the available 1.5-year results for ELIANA, expected survival rates at 2, 3, 4, and 5 years of follow-up were estimated for patients with r/r B-cell pALL treated with tisagenlecleucel. The methodology for the expert elicitation of these landmark survival estimates was adapted from the SHeffield ELicitation Framework (SHELF) [13–15]. The obtained survival estimates from multiple experts were combined with the empirical data from ELIANA to estimate long-term survival curves using parametric survival models. The different steps of the project are described in more detail here below. This study was conducted in accordance with the International Society for Pharmacoepidemiology Guidelines for Good Epidemiology Practices and was approved by Chesapeake Institutional Review Board.

### Expert selection

Experts were required to be board certified in oncology or hematology with at least five years of experience in pediatric medicine and prior experience with CAR-T therapy. A list of all ELIANA investigators was compiled. The known experts from the trial were asked to identify additional experts from among their acquaintances since clinicians with CAR-T therapy experience in the target population were expected to be limited considering the recent approval of tisagenlecleucel. From these experts, 19 were randomly selected and invited to join the study. The expected acceptance rate was between 25 and 50%, which would result in the recruitment of five to ten experts in this study. The sample size was designed to provide a sufficient diversity of opinion across the experts and the ability to confirm and validate shared views, which aligns with recommendations from SHELF [13–15].

Experts in pALL with experience in tisagenlecleucel and other CART-T cell therapies were invited to participate via email and were sent an information statement prior to enrollment, which outlined the purpose of the study, potential risks, and their rights and responsibilities. The study was double-blinded, meaning neither the experts nor the study sponsor were identified to each other. Experts were assured their identity would remain anonymous throughout the process and in any publication. All experts were identified from the ELIANA trial network, except for one case where a known expert suggested another expert with extensive CAR-T treatment experience in the target population. Information about the seven experts who participated is provided in Table 1.

**Table 1:** Overview of expert characteristics

Expert characteristics	*N* = 7 (%)
Years treating patients with pALL	
5–10	2 (29%)
10–20	3 (43%)
21+	2 (29%)
Approximate number of r/r pALL patients seen per month	
1–5	3 (29%)
6–10	1 (29%)
11–20	1 (14%)
21+	2 (29%)
Number of patients the expert has treated with any CAR T-cell therapy	
1–5	2 (29%)
6–10	2 (29%)
11–15	1 (14%)
25+	2 (29%)
Number of patients the expert has treated with tisagenlecleucel	
0	1 (14%)
1–5	3 (43%)
6–10	1 (14%)
11–15	2 (29%)

*pALL* pediatric acute lymphoblastic leukemia, *CAR* chimeric antigen receptor, *r/r* relapsed/refractory

### Elicitation of survival estimates beyond available ELIANA data at 2, 3, 4, and 5 years of follow-up

**Fig. 1:**
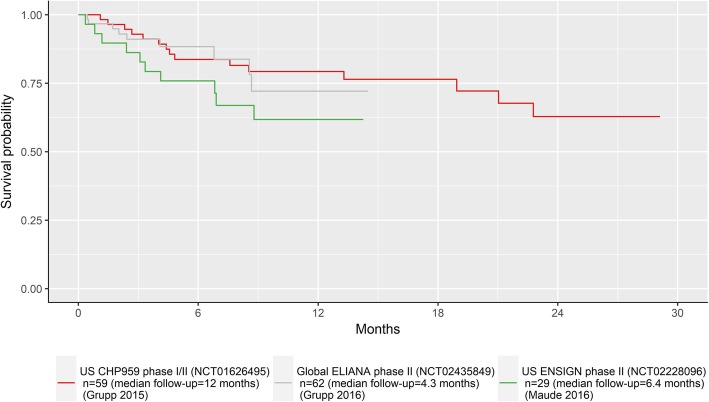
Tisagenlecleucel survival data presented to experts in evidence dossier

**Fig. 2:**
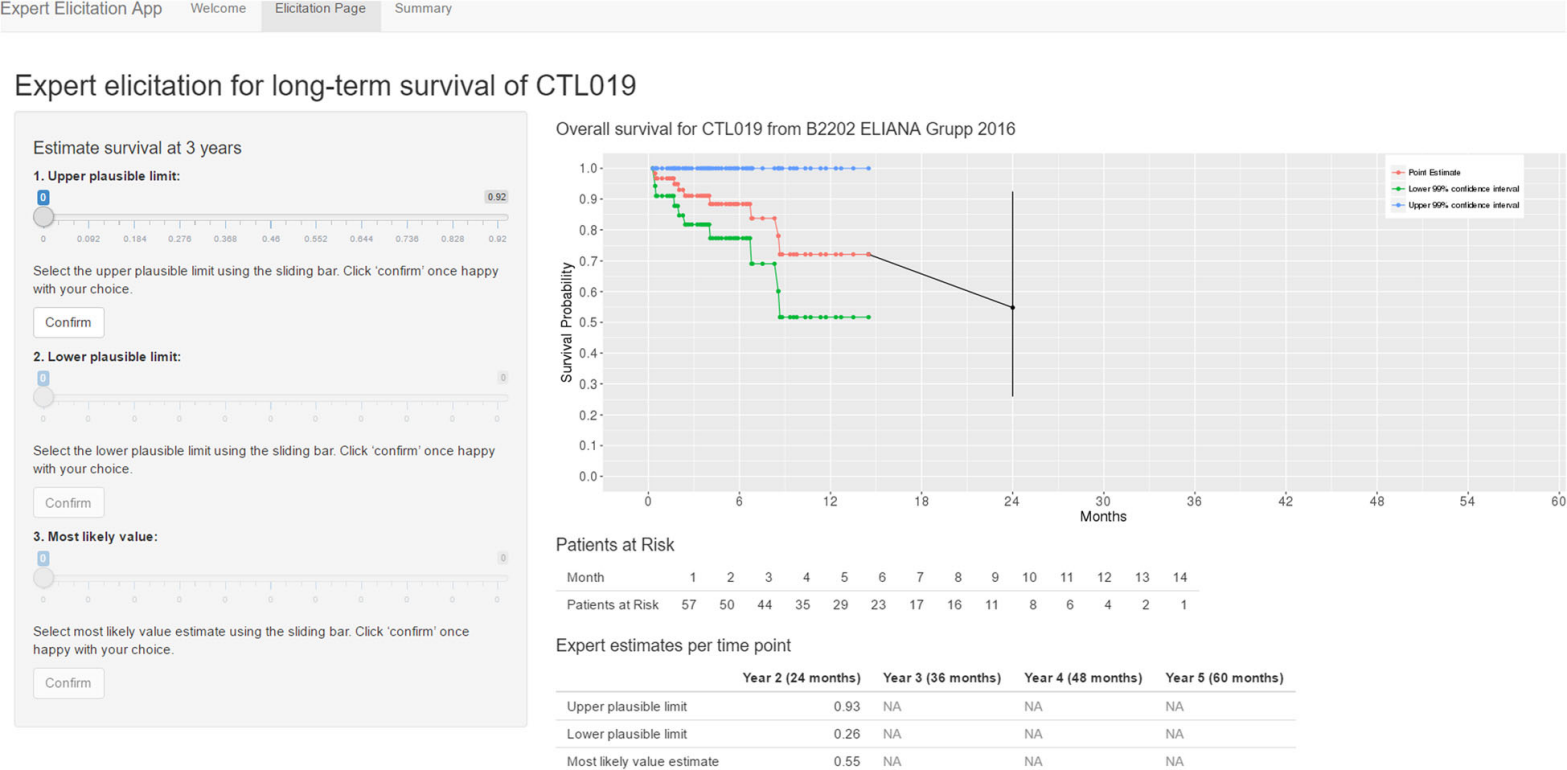
Web-based application for expert elicitation of overall survival between 2 to 5 years

During the interviews (May 12–30, 2017), an evidence dossier was reviewed to provide a common basis for expert judgments, which summarized the study purpose, tisagenlecleucel data for r/r pALL [12, 16, 17] (Fig. 1) and historical data for first-line pALL data and FDA approved interventions for r/r pALL (including stem cell transplant, clofarabine, and blinatumomab). The elicitation process and a practice exercise were also reviewed. Experts were guided through the elicitation using a web-based application (Fig. 2), which illustrated the survival from the ELIANA trial and the 99% confidence intervals [12]. SHELF guidance suggests that experts should be ‘almost certain that the quantity of interest lies within the plausible range (i.e. not physically impossible but extremely unlikely)’. In our study, the plausible limit was operationalized as the 99% confidence interval. For each time point, experts were asked to first estimate the upper plausible limit (UPL), followed by the lower plausible limit (LPL) and finally the most likely values (MLV) of survival. Experts used a sliding bar to select these values, which did not permit any illogical values for survival. Before confirming each value, experts were challenged to consider whether they were certain about their estimates in line with SHELF methodology. For example, experts were asked whether they favored estimates above or below their median estimates. Once experts confirmed their estimates for each time point, the results were plotted and summarized in a table. As final step, experts were presented with the graph of ELIANA data and their estimates at 2 to 5 years and were asked to confirm (or revise) their estimates.

### Estimation of extrapolated survival curves without expert information

The reported Kaplan-Meier (KM) curve for ELIANA was digitized (DigitizeIt; http://www.digitizeit.de/) and alternative parametric survival models were fitted to the corresponding discrete hazard data [18–20]. The following competing survival distributions were used: Weibull, Gompertz, and second-order fractional polynomials with power p_1_ = 0 or 1 and power p_2_ = − 1, − 0.5, 0, 0.5, or 1. These second-order fractional polynomial models can be considered extensions of the Weibull and Gompertz models, and allow arc- and bathtub shaped hazard functions. All analyses were performed in the Bayesian framework with non-informative prior distributions. Based on the obtained parameters for each of the survival models the corresponding survival curves were plotted up to 5 years of follow-up along including the 95% credible intervals. Additional detail is provided in Additional file 1**.**

The deviance information criteria (DIC) was used to compare the goodness-of-fit of the competing survival models [21]. DIC provides a measure of model fit to the data that penalizes model complexity. The model with the better trade-off between fit and parsimony has a lower DIC, where a difference of 3 to 7 points was considered meaningful [22].

All analyses were performed using a Markov Chain Monte Carlo (MCMC) method as implemented in the software package *Just Another Gibbs Sampler* (JAGS) (version 4.2.0), which were summarized in R (version 3.4.0). A first series of 20,000 iterations from the JAGS sampler were discarded as ‘burn-in’ and the inferences were based on 50,000 additional iterations using two chains. Convergence of the chains were confirmed by the Gelman-Rubin statistic.

### Consensus meeting

A consensus meeting was organized to summarize results of individual elicitations and to ask experts to judge what a *rational impartial observer* might reasonably believe, having seen their individual judgments and listened to their discussion. The aim was for the experts to reach agreement on a distribution representing a rational impartial view of their combined knowledge. An online web-chat was used to preserve anonymity, where a facilitator guided the discussion. Experts were presented with the ELIANA trial results, the individual expert elicitations and the two best fitting distributions to the observed ELIANA data (without expert information). Feedback from experts during the consensus meeting regarding the most appropriate distribution of survival was used to inform the selection of the final model. Each expert was asked whether they agreed with the modeled estimates as a reflection of the overall survival distribution. If they did not agree, they were asked whether estimates should be higher or lower, as well as the rationale for the estimates. All experts were invited to respond to individual reasoning and the process was repeated. Any experts who (still) disagreed with the estimates were asked to quantify their estimates. The qualitative feedback from experts regarding survival assumptions after five years was presented and experts were asked to share any further insights.

### Estimation of extrapolated survival curves with expert information

The elicited survival proportions from the experts at 2, 3, 4, and 5 years were formally integrated with the ELIANA data using a similar analytical approach as used for the estimation of survival curves solely based on ELIANA. For each expert, the elicited survival proportions along with the uncertainty at each time point were transformed into mortality probabilities (i.e. discrete hazards) for each interval corresponding to two subsequent time points. The survival proportion in the ELIANA trial at 1.5 years was used to calculate the discrete hazard for the first interval from 1.5 to 2 years. The set of seven expert-specific discrete hazard estimates for the 1.5 to 5-year time frame were added to the original set of discrete hazards from ELIANA and used to estimate the different survival curves according to Weibull, Gompertz and fractional polynomial models. This process was repeated for each of the seven experts and the results were subsequently combined by survival model to obtain average survival curves along with the 95% credible intervals reflecting the overall uncertainty across the elicited responses. The model selection process was consistent with the estimation of extrapolated survival curves without expert information. More detail is provided in Additional file 1.

### Comparison of survival curves with expert information to longer follow-up from ELIANA

Following the completion of the expert elicitation and evidence synthesis, longer follow-up from ELIANA was published where the infused patients had a median duration of follow-up of 24.2 months (range: 4.5–35.1 months) [23]. These results were compared to the estimates from experts to assess the accuracy of the expert estimates.

## Results

### Extrapolated survival curves without expert information

**Fig. 3:**
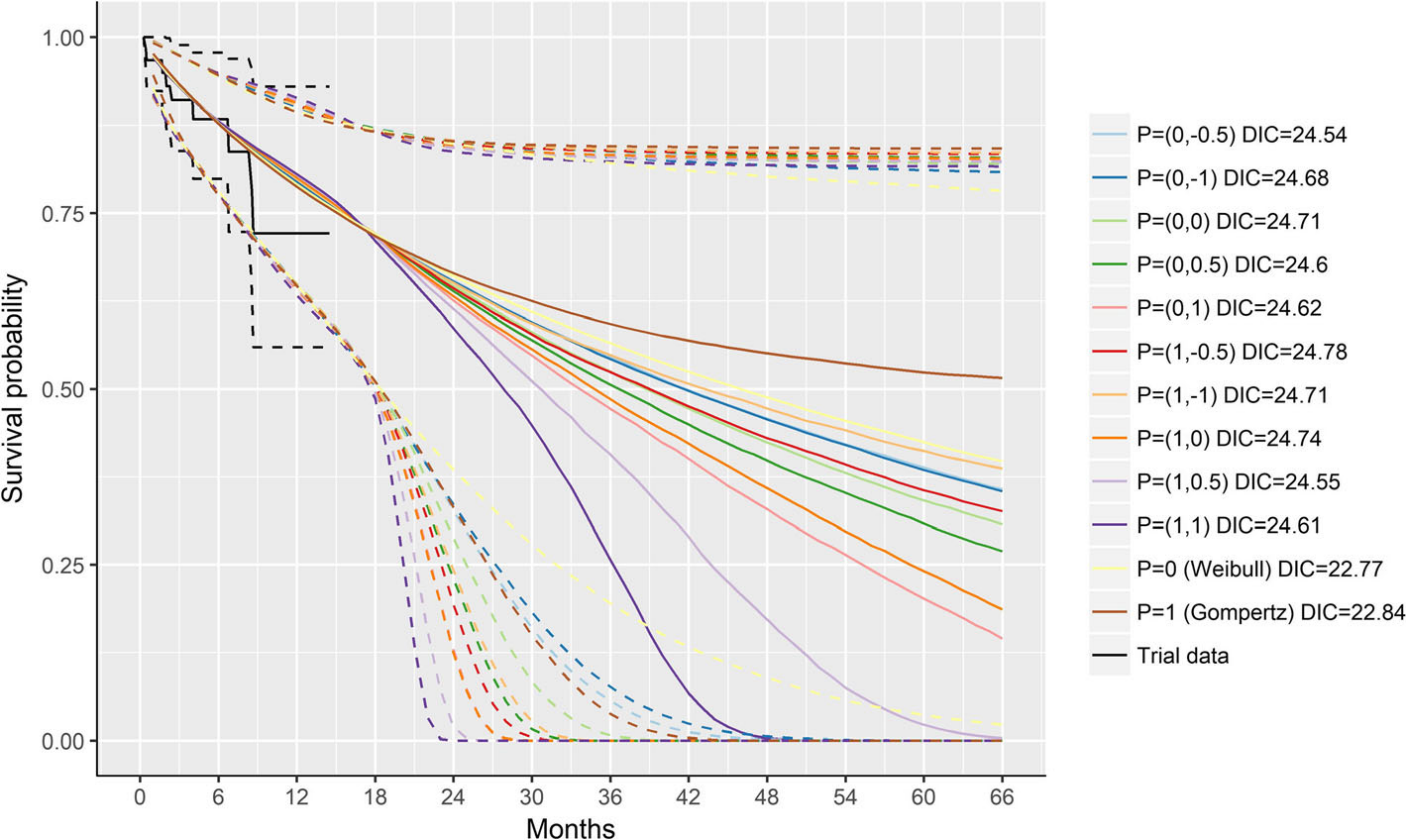
Modeled survival based on ELIANA trial data (1.5 years) without expert information. Solid lines represent point estimates and dashed lines the 95% credible intervals

Figure 3 illustrates the survival curves according to the different survival models estimated with data from ELIANA up to 1.5 years. The Gompertz and Weibull models resulted in the lowest DIC (i.e. 22.84 and 22.77, respectively), suggesting these models provided the best balance between fit and parsimony. The more complex second-order fractional polynomial models did not result in meaningful improvements (DICs ranging from 24.54 to 24.78). Importantly, these analyses illustrate the substantial variation in survival curves depending on the choice of model. The very wide 95% credible intervals highlight the substantial uncertainty in the extrapolated survival estimates caused by the limited follow-up data available from the ELIANA trial at the time of the analysis.

The curves corresponding to the Gompertz and Weibull model along with the observed ELIANA data were presented to experts during the consensus meeting (Additional file 2: Figure S1). Experts unanimously agreed that the Gompertz distribution reflected a consensus from the perspective of a rational impartial observer.

### Extrapolated survival curves with expert information

**Fig. 4:**
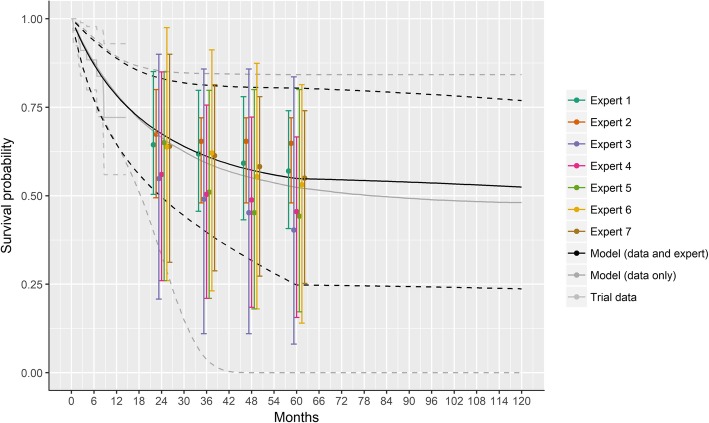
Modeled survival assuming Gompertz distribution based on ELIANA trial data with and without expert information. Note: Expert estimates are shifted slightly for each expert to help illustrate the specific overall survival values and ranges in their plausible limits at each time point; solid lines represent point estimates and dashed lines the 95% credible intervals

Figure 4 shows the overall survival data from ELIANA (up to 1.5 years) along with the survival proportions elicited from the individual experts. Point estimates from Experts 1, 6 and 7 were similar and clustered in the middle of the range, whereas Expert 2 was the most optimistic and Experts 3, 4, and 5 were less optimistic. Most experts showed a similar consistent decline in survival over time. However, Expert 2 showed almost constant survival, whereas Expert 5 estimated a sharp initial drop followed by a slowing of the decline. Experts 1 and 2 were most certain regarding their estimates, and the other experts showed similar levels of uncertainty.

The Gompertz and Weibull models provided the best trade-off between fit to the data and parsimony to estimate survival curves based on ELIANA in combination with the expert elicited survival proportions. Given the consensus among experts, the Gompertz model was selected as most appropriate. Figure 4 presents the survival curves according to the Gompertz model with and without expert information. The point estimates of the extrapolated survival proportions were comparable between these two analyses. However, the precision in the estimates was increased when expert information was incorporated as shown by the narrower 95% credible intervals. The survival at 2, 3, 4, and 5 years was estimated to be 67.5% (95% credible intervals: 49.5, 83.1%), 61.1% (39.5, 81.3%), 57.2% (31.5, 80.7%), and 54.9% (24.5, 80.5%), respectively.

### Comparison of survival curves with expert information to longer follow-up from ELIANA

**Fig. 5:**
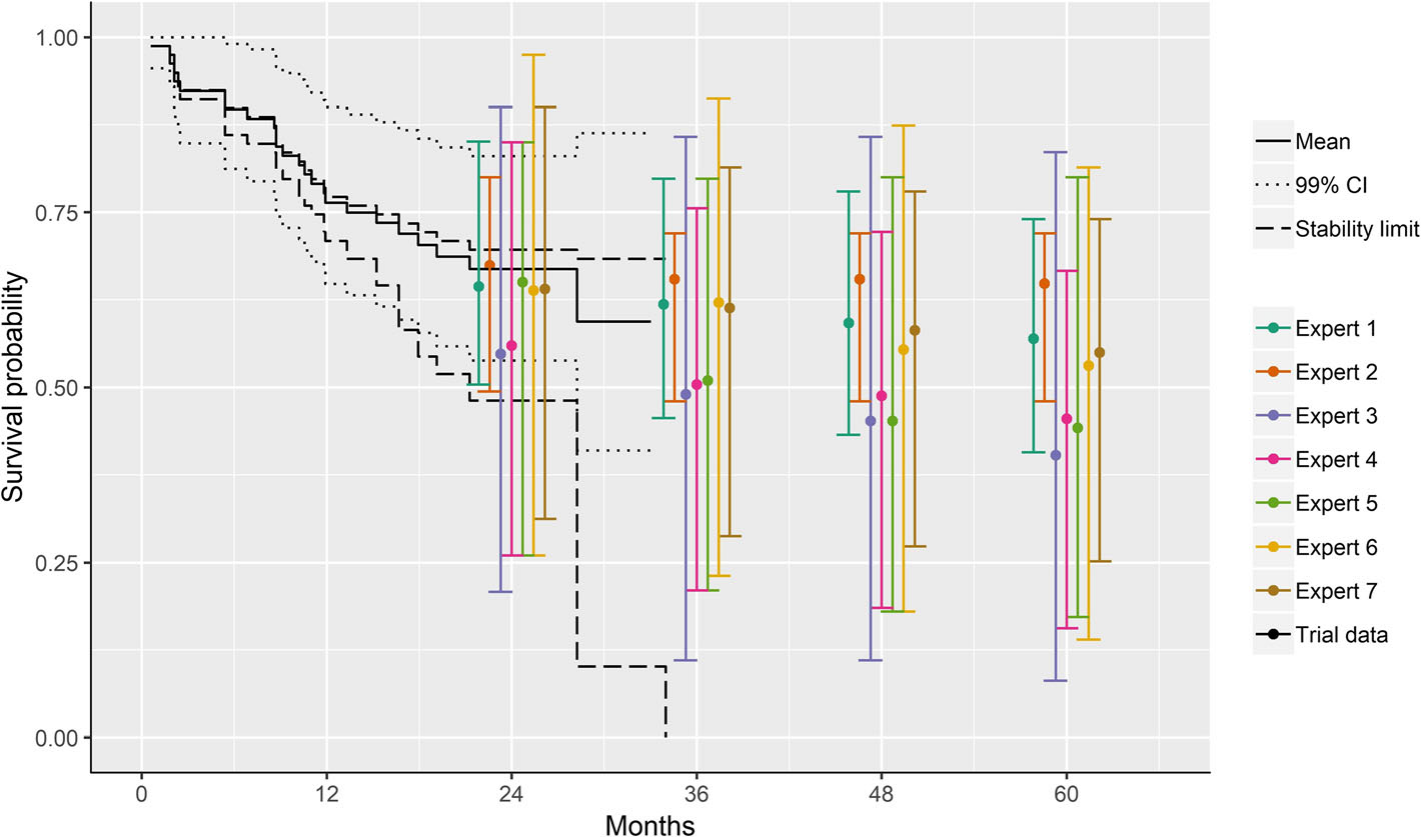
ELIANA trial data with expert information and updated analysis from ELIANA with longer follow-up

Figure 5 illustrates how the results incorporating expert opinion compare to the longer follow-up from ELIANA based on median duration of follow-up of 24.2 months (range: 4.5–35.1 months) [23]. Based on the longer term results, the survival at 24 months (66% [95% confidence interval, 54–76]) suggests the expert estimates were generally very close to the observed survival at that time point. Expert 2, the most optimistic, was almost exactly correct, whereas the other experts were more conservative in their estimates, particularly Experts 3 and 4 who were most pessimistic.

## Discussion

Given the increasing number of accelerated regulatory approvals for oncology treatments, health technology assessment agencies will be faced with the challenge of evaluating the value of new treatments with limited or immature overall survival data. To obtain useful extrapolated survival estimates, needed for cost-effectiveness evaluations, the available trial data needs to be supported with available external evidence, which may include expert opinion. In this paper, we presented an evidence synthesis method to integrate empirical survival data from a clinical trial with long-term estimates from a formal expert elicitation study.

The expert elicitation was performed using an established framework, i.e. SHELF, which is transparent and provides estimates of the most likely value for the parameters of interest along with estimates of uncertainty. This framework involves a rigorous process to select experts with clinically relevant experience in a double-blind manner. A comprehensive evidence dossier was developed to ensure a common basis for expert judgments, where experts had the opportunity to review and provide input. Experts were provided with background information on the process and training was provided with practice exercises. Since the SHELF method only provides methods for elicitation of individual time points or repeated measures rather than time-to-event outcomes, we developed a web-based application that would facilitate the elicitation and ensure experts were provided with immediate visual feedback regarding their elicitations, given that each new time point was dependent on the previous time point. Following the individual expert elicitations, consensus about the elicited long-term survival distribution from the perspective of rational impartial observer was achieved in a follow-up meeting, which allowed experts to interact. This process reflects a substantial improvement to standard practice for the development of develop a cost-effectiveness model, which often involves an informal validation of the selected model by a single expert.

In our case study survival data from the ELIANA trial (up to 1.5 years) was combined with expert-based survival estimates at 2, 3, 4, and 5 years for r/r pALL patients treated with tisagenlecleucel. All seven experts used for the elicitation exercise had extensive experience with pALL. However, their collective experience treating patients with tisagenlecleucel was limited given the early stage of the clinical trial at the time of the study. Survival estimates between 2 and 5 years showed a fair amount of variability between experts and their stated uncertainty was quite large in most cases. However, when these relatively uncertain elicited survival estimates were combined with the available data from ELIANA in the analysis, the precision of the extrapolated survival estimates dramatically increased in comparison to the extrapolated estimates without expert information. This highlights the power of the method presented in this paper [24–29]. However, this approach is motivated by the expectation that experts provide valuable information that is reasonably accurate. In our case study, updated results with longer follow-up from ELIANA suggest that experts were very close to the observed results at 24 months. Previous research has shown that experts tend to be optimistic; however, results at 24 months suggested six out of seven experts underestimated survival at 24 months. It will be important to evaluate longer term estimates in the future to access whether experts were optimistic given this intervention represented the first gene therapy as well as the first CAR-T therapy approved by FDA. Given the limited amount of follow-up at the time of the expert elicitation and the variation in the expert opinion, it may be worthwhile to also assess the most extreme expert estimates as alternative ‘low’ and ‘high’ scenarios in a cost-effectiveness analysis. Since there is still a large number of patients censored in the latest follow-up, it will be important to validate results again in the future based on longer term results.

During the consensus meeting experts agreed on the model using the Gompertz distribution, which provided a ‘middle ground’ given the distribution of expert estimates. Nonetheless, some differences in opinion were expressed in relation to survival beyond 5 years. Estimates of survival after 5 years were not elicited in the current exercise because pre*v*ious economic models for CAR-T therapy assumed all patients alive after 5 years were subsequently assumed to be long-term survivors [11] and were modeled based on general population all-cause risks of mortality adjusted for excess mortality reported related to pALL [30]. Two experts strongly agreed with long-term survival assumptions beyond 5 years. Two other experts agreed but acknowledged the possibility of relapse after 5 years due to differences between tisagenlecleucel and conventional therapy as well as the severity of the target population as compared to the general leukemia population. Finally, three experts believed that previous relapses and therapy burden related to prior transplants may affect long-term survival in the target population. These factors highlight the uncertainty regarding this new therapy in a difficult to treat population and may also explain differences between expert estimates between 2 and 5 years. It may be beneficial for future elicitations to include more experts as well as experts outside of the United States to ensure a more representative distribution. A larger number of experts from different areas may also facilitate more open discussion during the consensus meeting given that anonymity concerns may be less pronounced.

It is important to recognize that experts had limited information regarding the early results from this trial based on conference proceedings. Therefore, the level of detail available may have limited the experts’ understanding of the results. The risks associated with tisagenlecleucel are substantial, whereas specifics regarding adverse events were not fully described in the early results. Similarly, details regarding drop-outs, any deaths, and any subsequent treatments (allogeneic transplantation etc.), may be pertinent for experts. In future, presenting a swimmer plot to experts with patients categorized by response may provide more details to help provide more informed estimates. It should also be noted that the KM curve presented from ELIANA represents only the patients who were infused (i.e. time since infusion), whereas additional patients were included who discontinued prior to infusion (*n* = 18), who died or had adverse events between the time of enrollment and infusion (*n* = 10). This is important when interpreting the results, especially when comparing these estimates to other interventions where infusion is not a required step.

Although the SHELF framework was used, since there are no existing templates for time-to-event outcomes, the process was simplified in some respects. There are alternative approaches to elicit uncertainty within SHELF, such as ‘quantiles’ or ‘roulette’ (also known as ‘histogram’) methods. We only asked experts to assess the upper and lower values and the most likely values, rather than asking them to estimate the quartiles for each time point, which would require an estimate of the probability that their estimated value lies within each interval. In our view, there was a risk of overcomplicating the exercise given the number of time points required, possibly jeopardizing the quality of the estimates. A study by Grigore et al. [3] identified the challenge of selecting the most appropriate elicitation method and found that their results were not sensitive the choice between histogram or the ‘hybrid’ method. However, additional research would be helpful to better characterize the shape of these distributions based on other methods.

**Fig. 6:**
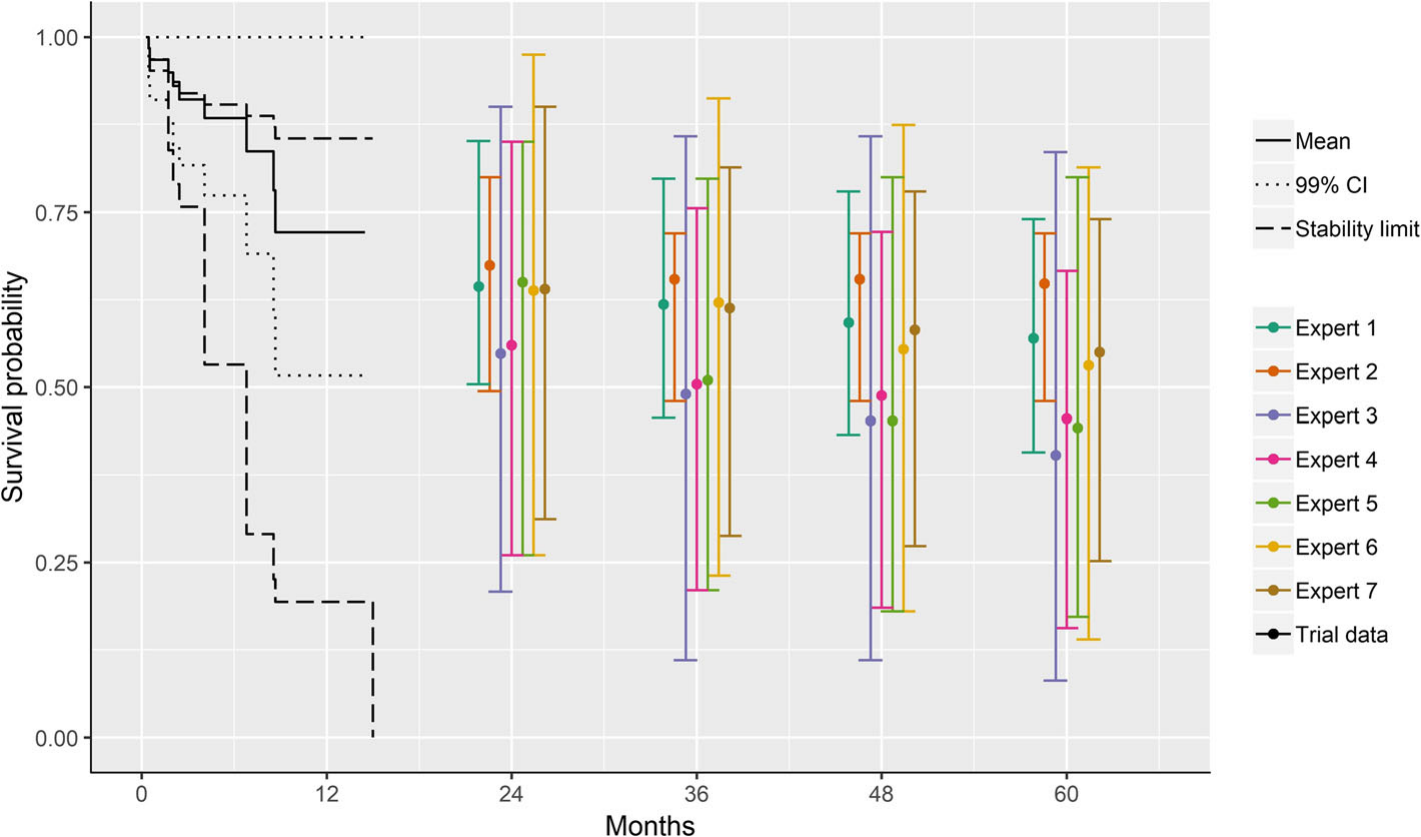
ELIANA trial data (1.5 years) with low (pessimistic) and high (optimistic) curves evaluating ‘stability’ and expert information

We elicited survival estimates based on a KM curve, which requires a sufficient understanding of the time-to-event analyses. In our case study, this is particularly important given the limited follow-up time available in the initial analysis. Given such a small sample of children and young adults who have failed two prior regimens is likely to be one of the most challenging populations to predict survival, since one death can have a dramatic effect on the survival estimates. It is possible that experts did not fully appreciate how censoring is handled in a KM curve and were therefore unduly influenced by the flat tail of the curve presented. In a previous study, 82% of clinicians’ correctly interpreted relative risk, however only 11% understood KM curves and could interpret the 95% confidence intervals and statistical significance [26]. Therefore, it may be helpful to restrict the tail of the KM curve when only 10–20% of the original sample are at risk (or when sample is less than 10) as has been recommended previously [27, 28] to avoid over interpreting the ‘tail’. It also appears that the some experts were not clear on the definitions of the upper and lower bounds. The elicitation aimed to identify the plausible range of values, and the bounds were meant to reflect the extremes of this range. While most experts provided wide intervals, two experts had lower bounds of approximately 50% at 5 years of follow up. It seems unlikely that they truly believed that it was impossible to have survival probabilities less than this, and therefore this lower bound may have been interpreted differently by these experts. In future, it may be helpful to illustrate the upper and lower extremes of a KM curve, rather than simply the confidence intervals. This approach, to substitute the censors with non-events (optimistic curve) or events (pessimistic curve), has been proposed to help illustrate the ‘stability’ of the results given the available follow-up [29]. Figure 6 illustrates these optimistic and pessimistic curves for the ELIANA data presented to experts, which shows that the pessimistic curve is below the lower confidence interval as well as further from the observed values as compared to the optimistic curve. This may help clinicians to visualize how uncertain results are given the limited follow-up. Ultimately, additional research is required to evaluate the best approach to elicit time-to-event data to ensure that results are as realistic as possible. Moreover, it is important to assess how much follow-up is sufficient to inform reliable predictions as well as decision-making. Even in the updated data cut, there are still 54 censored patients. Examining the optimistic and pessimistic curves for this dataset shows there is less uncertainty as compared to the initial cut of data; however it is still possible for long-term survival to be to be less optimistic than that predicted by the experts. This underscores the need to update analyses based on further follow-up before definitive conclusions are drawn. Ultimately, a validation cohort was not evaluated within the current study, which reflects a limitation of the study.

There is no standard methodology to combine expert opinion with clinical data, though this is an area of active research [2]. Our methods, which consider expert opinion as additional data, are similar to that employed by Guyot et al. 2017, who included external evidence from an observational database [5]. The model we have developed improves on existing survival models by systematically integrating external evidence from experts, which could be generalized to any cost-effectiveness analysis. The current synthesis used elicited survival proportions from the experts, which were analyzed using the observed trial data augmented with the additional expert information included as an artificial data set. This approach ensured that expert estimates were grounded in relation to the observed data in the ELIANA trial. Adding expert information to the model validated the model choice and reduced the amount of uncertainty when compared to the model without expert information. It is important to highlight that the estimates from each expert were modelled separately, and that the overall estimate reflects a combined overall distribution. This approach avoids pooling or a model averaging, which would provide narrower intervals around the mean. Therefore, in our approach, adding additional experts, does not lead to more precise estimates. However, it may also be feasible to develop a hierarchical model which combines the trial and expert data, by allowing each source to provide parameter estimates from a common distribution. We are currently investigating methodologies for of time-to-event models that will account for sources of information, to ensure that increasing number of experts does not increase the parameter uncertainty.

To ensure that the exercise was intuitive for experts it was necessary to elicit survival proportions over time. This meant that we had to relate the survival estimates and related uncertainty to the underlying hazard to estimate the parameters of the log-hazard function for the alternative fractional polynomial models. In our model, the expert elicited survival proportions and uncertainty were assumed to follow a normal distribution, which is a reasonable assumption (despite being bounded by 0 and 1) because the elicited probabilities fell within the middle of the range of possible values, and the elicited ranges were symmetric around the MLV. As an alternative to the normal distribution, it may be possible to use a beta distribution to characterize the survival distributions at each time point for each expert. Finally, these methods could be extended beyond fractional polynomial models to spline models proposed by Royston and Parmer [31] used by Hettle et al. [11].

## Conclusions

This study provides an example of how expert opinion can be elicited and combined with observed survival data from trials in a transparent, formal, and reproducible manner, to ensure that projected long-term survival can be integrated in cost-effectiveness models and is clinical plausible. This method provides a meaningful improvement over the standard approaches to incorporate expert opinion in cost-effectiveness modeling, which frequently involves a post-hoc validation of extrapolated survival curves by a single expert. Based on ELIANA trial data and expert opinion, it is predicted that more than half of the pALL patients treated with tisagenlecleucel will be alive at five years of follow-up. However, additional follow-up is required to ensure that estimates elicited from experts improve the plausibility of the predicted survival curves.

## Additional files

Additional file 1: Time-to-even methodology. (DOCX 49 kb)

Additional file 2: **Figure S1** Modeled survival based on ELIANA without expert information: A) Gompertz distribution and B) Weibull distribution. (TIF 358 kb)

## Data Availability

The datasets generated during and/or analysed during the current study are available from the corresponding author on reasonable request.

## Abbreviations

CAR-T: Chimeric antigen receptor T-cell
DIC: Deviance information criterion
FDA: Food and Drug Administration
JAGS: Just Another Gibbs Sampler
KM: Kaplan-Meier
LPL: Lower plausible limit
MCLC: Markov Chain Monte Carlo
MLV: Most likely values
NICE: National Institute for Health Care and Excellence
pALL: Pediatric acute lymphoblastic leukemia
r/r: Relapsed/refractory
SHELF: SHeffield ELicitation Framework
UPL: Upper plausible limit

## Footnotes

1
https://www.pharma.us.novartis.com/sites/www.pharma.us.novartis.com/files/kymriah.pdf
