# Supplementary material for: Integrating expert opinion with clinical trial data to extrapolate long-term survival: a case study of CAR-T therapy for children and young adults with relapsed or refractory acute lymphoblastic leukemia
Source: BMC Med Res Methodol. 2019 Sep 2;19:182. doi: 10.1186/s12874-019-0823-8 (PMC6721254; doi:10.1186/s12874-019-0823-8)
Supplement: Supplementary file 1 — Time-to-even methodology. (DOCX 49 kb) [file 12874_2019_823_MOESM1_ESM.docx]

# Additional file 1: Time-to-event methodology

*Network meta-analysis model for time-to-event data*

Survival or time-to-event is a function of the underlying hazard of the event of interest (i.e. progression or death) at a given point in time. When survival data is available at multiple time points such as in KM curves, fractional polynomial models were used to synthesize the underlying hazard functions [19, 20]

(1)

where $h_{jkt}$ reflects the underlying hazard rate in trial *j* for intervention *k* at time point *t* and is now described as a function of time *t* where $p=\{0,1\}\text{ and }t^{0}=\text{log(t)}$ with treatment and study specific scale and shape parameters $\beta_{0jk}$and $\beta_{1jk}$. If$\beta_{1jk}$ equals 0, a constant log hazard function is obtained, reflecting exponentially distributed survival times. If $\beta_{1jk}$≠ 0 and *p*=1 a linear hazard function is obtained which corresponds to a Gompertz survival function. If $\beta_{1jk}$ ≠ 0 and *p*=0 a Weibull hazard function is obtained.

For additional flexibility, this first order fractional polynomial model can be generalized to a 2^nd^ order fractional polynomial model, for greater model flexibility and probably better fit to the data [19].

(2)

*Likelihood distribution for time-to-event model*

KM curves can be divided into *q* consecutive intervals over the follow-up period: [*t*_1_, *t*_2_], (*t*_2_, *t*_3_], …, (*t_q_*, *t_q_*_+1_] with *t*_1_=0. For each time interval *m*=1,2,3,…,*.q,* an algorithm was used to calculate the patients at risk at the beginning of that interval and incident number of deaths given the survival proportions [20]. A binomial likelihood distribution of the incident events for every interval can be described according to: where *r_jkt_* is the observed number of events in the m^th^ interval ending at time point *t_m_*_+1_ for treatment *k* in study *j*. *n_jkt_* is the number of subjects at risk just before the start of that interval adjusted for the subjects censored in the interval. *p_jkt_* is the corresponding underlying event probability. When the time intervals are relatively short, the hazard rate *h_jkt_* at time point *t* for treatment *k* in study *j* can be assumed to be constant for any time point within the corresponding m^th^ time interval. The hazard rate corresponding to *p_jkt_* for the m^th^ interval can be standardized by the unit of time used for the analysis (e.g. months) according to where ∆*t_jkt_* is the length of the interval. For the model estimation, we assigned this underlying hazard to time point *t_m_*_+1_.

The expert-elicited data did not provide information on either the number of patients at risk, or the probability of experiencing an event in each time interval, which precludes the use of a binomial likelihood. The experts provided estimates of survival at 2, 3, 4, and5 years post follow-up. The survival is a function of the cumulative hazard to the time of interest. For data related to survival, a Normal likelihood was employed. The survival at time t is defined as S(t) ~ N(𝜇_S(t)_,σ^2^_S(t)_). The variance of this normal random variable is defined from the width of the interval provided by the expert. The estimated survival is then used to identify the probability of survival during the preceding interval: (1) $p_{m+1}=1-\mu_{S(t_{m+1})}/\mu_{S(t_{m})}$and (2) $h_{m}=-\ln(1-p_{m})/\Delta t_{m}$ .

*Prior distributions for time-to-event models*

Non-informative prior distributions were used for all models

The prior distributions for model 1 are:

For model 2 the prior distributions for the study effects are:

*Incorporation of elicited survival proportions from experts*

For each time interval *m* =1,2,3,…,*.r* the discrete hazard was calculated using the following relationships: (1) $p_{m+1}=1-S(t_{m+1})/S(t_{m})$and (2) $h_{m}=-\ln(1-p_{m})/\Delta t_{m}$where $S(t_{m})$ is the survival at time point corresponding to interval *m*, $p_{m}$reflects the probability of death within interval *m,* $h_{m}$is the hazard within interval *m*, and $\Delta t_{m}$is the length of the interval.
