# Supplementary figures and images for: Integrating expert opinion with clinical trial data to extrapolate long-term survival: a case study of CAR-T therapy for children and young adults with relapsed or refractory acute lymphoblastic leukemia
Source: BMC Med Res Methodol. 2019 Sep 2;19:182. doi: 10.1186/s12874-019-0823-8 (PMC6721254; doi:10.1186/s12874-019-0823-8)

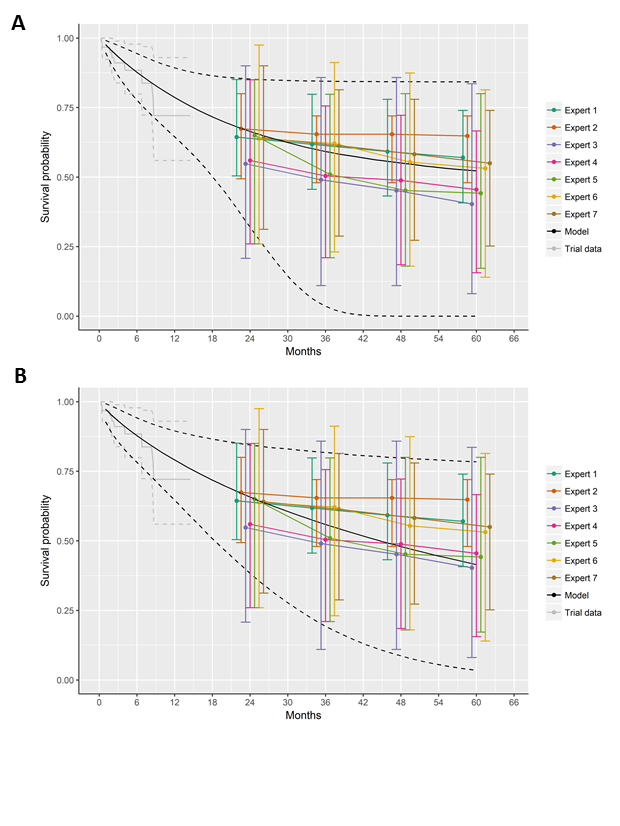

Supplement: Supplementary file 2 — Figure S1 Modeled survival based on ELIANA without expert information: A) Gompertz distribution and B) Weibull distribution. (TIF 358 kb) [file 12874_2019_823_MOESM2_ESM.tif]
